# Hybrid Quantum Protocols for Secure Multiparty Summation and Multiplication

**DOI:** 10.1038/s41598-020-65871-8

**Published:** 2020-06-04

**Authors:** Kartick Sutradhar, Hari Om

**Affiliations:** Indian Institute of Technology (ISM) Dhanbad, Department of Computer Science and Engineering, Dhanbad, 826004 India

**Keywords:** Engineering, Computer science

## Abstract

The summation and multiplication are two basic operations for secure multiparty quantum computation. The existing secure multiparty quantum summation and multiplication protocols have (*n*, *n*) threshold approach and their computation type is bit-by-bit, where *n* is total number of players. In this paper, we propose two hybrid (*t*, *n*) threshold quantum protocols for secure multiparty summation and multiplication based on the Shamir’s secret sharing, SUM gate, quantum fourier transform, and generalized Pauli operator, where *t* is a threshold number of players that can perform the summation and multiplication. Their computation type is secret-by-secret with modulo *d*, where *d*, *n* ≤ *d* ≤ 2*n*, is a prime. The proposed protocols can resist the intercept-resend, entangle-measure, collusion, collective, and coherent quantum attacks. They have better computation as well as communication costs and no player can get other player’s private input.

## Introduction

The secure multiparty quantum computation is an essential component in quantum cryptography. The summation and multiplication are two basic operations for secure multiparty quantum computation. The secure multiparty quantum summation and multiplication include a list of secrets, which is shared among a set of players and the players jointly perform summation or multiplication without revealing their secrets. The classical summation and multiplication protocols cannot provide the unconditional secure communications. However, the quantum summation and multiplication protocols can provide the unconditional security as they are based on the principles of quantum mechanics like quantum correlation^1^, entanglement for bipartite system^2^, Heisenberg XYZ model^3^. In 2007, Du *et al*.^4^ discussed a secure multiparty quantum addition modulo *n* + 1 protocol based on the non-orthogonal states, where *n* is total number of players. In 2010, Chen *et al*.^5^ introduced a secure multiparty quantum addition modulo 2 protocol based on multi-particle entangle. In 2014, Zhang *et al*.^6^ presented a protocol with addition modulo 2 based on both polarization of a single photon. In 2010, a three-party quantum addition modulo 2 protocol was discussed by Zhang *et al*.^7^. These protocols have some limitations, for example, they cannot perform addition correctly if one player is dishonest as these protocols have (*n*, *n*) threshold approach, and the modulo of these protocols is very small. They have high communication and computation costs due to the bit-by-bit computation. In 2015, Shi *et al*.^8^ discussed a secure multiparty quantum summation and multiplication protocol. This protocol is efficient, but it has (*n*, *n*) threshold approach. In 2017, Shi and Zhang^9^ introduced a two-party quantum protocol for summation. This protocol cannot perform summation correctly if one party is dishonest. In the same year, a multiparty quantum summation modulo 2 protocol was discussed by Zhang *et al*.^10^. This protocol is efficient but its modulo is too small. Then, Liu *et al*.^11^ discussed a secure multiparty quantum summation protocol based on two particle Bell States. This protocol is efficient but its modulo is 2 and it has (*n*, *n*) threshold approach. In 2018, Yang and Ye^12^ introduced a secure multiparty quantum protocol for summation based on the quantum Fourier transform. The computation type of this protocol is secret-by-secret, but it has (*n*, *n*) threshold approach. Recently, Jiao *et al*.^13^ have discussed a secure multiparty quantum summation and multiplication protocol with mutually unbiased bases. This protocol is efficient, but the computation type of this protocol is secret-by-secret and it has (*n*, *n*) threshold approach. Most of the existing secure multiparty summation and multiplication protocols have (*n*, *n*) threshold approach and bit-by-bit computation type. Further, they are not practically feasible as they require high communication as well as computation costs.

In this paper, we propose two hybrid (*t*, *n*) threshold quantum protocols for secure multiparty summation and multiplication. In order to incorporate the advantages of both quantum and classical multiparty summation and multiplication, we apply the quantum methods in a secure multiparty computation. The novelty of the proposed work can be summarized as follows. Our protocols have (*t*, *n*) threshold approach, where *t* players can perform the quantum summation and multiplication systematically and efficiently without revealing their privacy. They require less communication and computation costs as their computation type is secret-by-secret. Further, our proposed protocols possess all the benefits(i.e., they use qudit instead of qubit, do not require the secret information to be passed through the transmitted particles, do not require to perform entanglement measurement, and the quantum attacks cannot be performed) of the existing secure multiparty quantum summation and multiplication.

## Preliminaries

Here, we introduce the Shamir’s secret sharing, quantum state, SUM gate, quantum Fourier transform (*QFT*), and generalized Pauli operator, which will be used in our work.

### Shamir’s secret sharing

In the Shamir’s secret sharing, there is a set of players ${\mathscr{P}}=\{{P}_{1},{P}_{2},\ldots ,{P}_{n}\}$ and a dealer^14^. This secret sharing scheme consists of two phases: secret sharing and secret reconstruction. In secret sharing phase, the dealer shares a secret among *n* players using a (*t* − 1) degree polynomial *f*(*y*); each player knows only his share. In secret reconstruction phase, the *t* players reconstruct the secret using the Lagrange interpolation. The Lagrange Interpolation is defined as follows.

1 $$f(y)=\mathop{\sum }\limits_{k=1}^{t}\,f({y}_{k})\prod _{1\le j\le t,j\ne k}\,\frac{y-{y}_{j}}{{y}_{k}-{y}_{j}}$$

For *y* = 0, Eq.(1) can be written as follows.

2 $$\begin{array}{rcl}f(0) & = & \mathop{\sum }\limits_{k=1}^{t}\,f({y}_{k})\prod _{1\le j\le t,j\ne k}\,\frac{-{y}_{j}}{{y}_{k}-{y}_{j}}\\  & = & \mathop{\sum }\limits_{k=1}^{t}\,f({y}_{k})\prod _{1\le j\le t,j\ne k}\,\frac{{y}_{j}}{{y}_{j}-{y}_{k}}\end{array}$$

### Quantum State

In quantum computing, the *d*-level quantum state is defined as follows^15^.

3 $$|{\phi }\rangle =\mathop{\sum }\limits_{l=0}^{d-1}\,{C}_{l}|l\rangle $$

It must satisfy ${\sum }_{l=0}^{d-1}\,|{C}_{l}{|}^{2}=1$, where *C*_*l*_ represents complex number.

### SUM gate

In quantum computing, the SUM gate is defined as follows^16^.

4 $$SUM(|u\rangle ,|v\rangle )=(|u\rangle ,|u+v\,{\rm{mod}}\,d\rangle ),$$

where |*u*〉 and |*v*〉 denote control and target particles, respectively, and $u,v\in \{0,1,\ldots ,d-1\}$.

### Quantum Fourier Transform (*QFT*)

The discrete Fourier transform is the foundation of *QFT*. The *d*-level *QFT* is defined as follows^8^.

5 $$QFT|p\rangle =\frac{1}{\sqrt{d}}\mathop{\sum }\limits_{l=0}^{d-1}\,{\omega }^{p.l}|q\rangle $$

where, *ω* represents *e*^2*πi*^.

### Generalized Pauli operator

In quantum computing, the *d*-level generalized Pauli operator is defined as follows^17^.

6 $${U}_{\gamma ,\delta }=\mathop{\sum }\limits_{l=0}^{d-1}\,{\omega }^{l.\delta }|l+\gamma \rangle \langle l|,$$

where, $$\gamma ,\delta \in \{0,1,\ldots d-1\}$$.

## Proposed Protocols

In this section, we propose two hybrid (*t*, *n*) threshold secure multiparty quantum summation and multiplication protocols. Let *X* and *Y* have two secrets *a* and *b*, respectively, and want to perform (*a* + *b*) or (*a* × *b*) without revealing their secrets. In these proposed protocols, we assume that the set of players ${\mathscr{P}}=\{{P}_{1},{P}_{2},\ldots ,{P}_{n}\}$, a qualified subset ${\mathbb{S}}=\{{P}_{1},{P}_{2},\ldots ,{P}_{t}\}$, each qualified subset containing *t* players and player *P*_1_ is an initiator.

### Hybrid secure multiparty quantum summation protocol

Here, we discuss our proposed hybrid (*t*, *n*) threshold secure multiparty quantum summation, whose procedure is given as follows.

**Step 1:** The *X* and *Y* choose two different polynomials $f(y)=a+{c}_{1}y+{c}_{2}{y}^{2}+\ldots +{c}_{t-1}{y}^{t-1}\,{\rm{mod}}\,d$ and $g(y)=b+{\bar{c}}_{1}y+{\bar{c}}_{2}{y}^{2}+\ldots +{\bar{c}}_{t-1}{y}^{t-1}\,{\rm{mod}}\,d$, where ${c}_{1},{c}_{2},\ldots ,{c}_{t-1}$ and ${\bar{c}}_{1},{\bar{c}}_{2},\ldots ,{\bar{c}}_{t-1}$ are coefficients and *a* and *b* are the secrets of *X* and *Y*, respectively. Then, they compute the classical shares *f*(*y*_*i*_) and *g*(*y*_*i*_), respectively, using the Shamir’s Secret Sharing^14^ and distribute these shares among the set of players ${\mathscr{P}}=\{{P}_{1},{P}_{2},\ldots ,{P}_{n}\}$. Player *P*_*i*_, $i=1,2,\ldots ,n$, possesses only his shares *f*(*y*_*i*_) and *g*(*y*_*i*_).

**Step 2:** Player *P*_*i*_, $i=1,2,\ldots ,$ computes $h({y}_{i})=f({y}_{i})+g({y}_{i})\,{\rm{mod}}\,d$ and keeps *h*(*y*_*i*_) secret.

**Step 3:** Player *P*_*k*_, $k=1,2,\ldots ,t$, computes the shadow of his shares, denoting as *A*_*k*_, as follows.

7 $${A}_{k}=h({y}_{k})\prod _{1\le j\le t,j\ne k}\,\frac{{y}_{j}}{{y}_{j}-{y}_{k}}\,{\rm{mod}}\,d$$

**Step 4:** Initiator *P*_1_ prepares *t* single qudit particles |0〉_1_, |0〉_2_, …, |0〉_*t*_ and applies *QFT* on the first particle |0〉_1_. The resultant state |*ϕ*_1_〉 is given as follows.

8 $$|{\phi }_{1}\rangle =(QFT{|0\rangle }_{1})=\frac{1}{\sqrt{d}}\mathop{\sum }\limits_{l=0}^{d-1}\,{|l\rangle }_{1}$$

Here, |*ϕ*_1_〉 and |0〉_*k*_’s, $k=2,3,\ldots ,t$, are control and target qudits, respectively.

**Step 5:** Player *P*_1_ performs (*t* − 1) SUM operations to produce the entangled state |*ϕ*_2_〉 as follows.

9 $$|{\phi }_{2}\rangle =\frac{1}{\sqrt{d}}\mathop{\sum }\limits_{l=0}^{d-1}\,{|l\rangle }_{1}{|l\rangle }_{2}\ldots {|l\rangle }_{k}.$$

Initiator *P*_1_ sends the particle |*l*〉_*k*_ to each player *P*_*k*_, $k=2,3,\ldots ,t$.

**Step 6:** Each player *P*_*k*_, $k=2,3,\ldots ,t$, applies the *QFT* operation on his particle |*l*〉_*k*_ and then executes the generalized Pauli operator ${U}_{{A}_{k},0}$, $k=1,2,\ldots ,t$. The quantum state |*ϕ*_2_〉 is evolved as the quantum state |*ϕ*_3_〉, which is obtained as follows:

10 $$\begin{array}{rcl}|{\phi }_{3}\rangle  & = & {U}_{{A}_{1}\mathrm{,0}}QFT\otimes {U}_{{A}_{2}\mathrm{,0}}QFT\otimes \ldots \otimes {U}_{{A}_{k}\mathrm{,0}}QFT|{\phi }_{2}\rangle \\  & = & {d}^{-\frac{t+1}{2}}\sum _{0\le {m}_{1},{m}_{2},\ldots ,{m}_{k} < d\mathrm{,\ }{m}_{1}+{m}_{2}+,\ldots ,+{m}_{k}\mathrm{=0}\,mod\,d}\,|{m}_{1}+{A}_{1}\rangle |{m}_{2}+{A}_{2}\rangle \ldots |{m}_{k}+{A}_{k}\rangle \end{array}$$

**Step 7:** In computational basis, each player *P*_*k*_, $k=2,3,\ldots ,t$, measures his particle $|{m}_{k}+{A}_{k}\rangle$ and broadcasts his measurement result *m*_*k*_ + *A*_*k*_, $k=1,2,\ldots ,t$.

**Step 8:** Finally, they jointly compute the summation by adding their measurement results. The summation of the secrets is computed as: $(a+b)={\sum }_{k=1}^{t}\,{m}_{k}+{A}_{k}\,{\rm{mod}}\,d$.

### Hybrid secure multiparty quantum multiplication protocol

Here, we discuss our proposed quantum protocol for secure multiparty multiplication, whose procedure is given as follows.

**Step 1:** Initially, *X* and *Y* select two different polynomials $f(y)=a+{c}_{1}y+{c}_{2}{y}^{2}+\ldots +{c}_{t-1}{y}^{t-1}\,\mathrm{mod}\,d$ and $g(y)=b+{\bar{c}}_{1}y+{\bar{c}}_{2}{y}^{2}+\ldots +{\bar{c}}_{t-1}{y}^{t-1}\,{\rm{mod}}\,d$, where *a* and *b* are the secrets of *X* and *Y*, ${c}_{1},{c}_{2},\ldots ,{c}_{t-1}$ and ${\bar{c}}_{1},{\bar{c}}_{2},\ldots ,{\bar{c}}_{t-1}$ are coefficients of polynomials *f*(*y*) and *g*(*y*), respectively. Then, they compute the classical shares *f*(*y*_*i*_) and *g*(*y*_*i*_), respectively, and distribute them among *n* players. Player *P*_*i*_, $i=1,2,\ldots ,n$, knows his shares *f*(*y*_*i*_) and *g*(*y*_*i*_) only.

**Step 2:** Player *P*_*i*_, $i=1,2,\ldots ,n$, computes $h{\prime} ({y}_{i})=f({y}_{i})\times g({y}_{i})\,{\rm{mod}}\,d$ and shares $h{\prime} ({y}_{i})$ among *n* players using new random polynomial ${z}_{i}(x)=h{\prime} ({y}_{i})+{\beta }_{1}x+{\beta }_{2}{x}^{2}+\ldots +{\beta }_{t-1}{x}^{t-1}\,{\rm{mod}}\,d$. The total polynomial, denoted as *T*_*i*_, is computed by each player *P*_*i*_, $i=1,2,\ldots ,n$, using the Vandermonde matrix^18^. Player *P*_*i*_, $i=1,2,\ldots ,n$, knows *T*_*i*_ only.

**Step 3:** Each player *P*_*k*_, $k=1,2,\ldots ,t$, computes the shadow *B*_*k*_ of his share as follows.

11 $${B}_{k}={T}_{k}\prod _{1\le j\le t,j\ne k}\,\frac{{x}_{j}}{{x}_{j}-{x}_{k}}\,{\rm{mod}}\,d$$

**Step 4:** Initiator *P*_1_ prepares *t* single qudit particles ${|0\rangle }_{1},{|0\rangle }_{2},\ldots ,{|0\rangle }_{t}$ and applies *QFT* on the first particle 0_1_. The resultant state |Φ_1_〉 is given as follows.

12 $$|{\Phi }_{1}\rangle =(QFT{|0\rangle }_{1})=\frac{1}{\sqrt{d}}\mathop{\sum }\limits_{r=0}^{d-1}\,{|r\rangle }_{1}.$$

Here, |Φ_1_〉 denotes control qudit and |0〉_*k*_’s, $k=2,3,\ldots ,t)$, denote target qudits.

**Step 5:** Initiator executes (*t* − 1) SUM operations to get the entangled state |Φ_2_〉, as follows.

13 $$|{\Phi }_{2}\rangle =\frac{1}{\sqrt{d}}\mathop{\sum }\limits_{r=0}^{d-1}\,{|r\rangle }_{1}{|r\rangle }_{2}\ldots {|r\rangle }_{k}.$$

Initiator *P*_1_ sends the particle |*r*〉_*k*_ to each player *P*_*k*_, $k=2,3,\ldots ,t$.

**Step 6:** Each player *P*_*k*_, $k=2,3,\ldots ,t$, applies *QFT* on his particle |*r*〉_*k*_, followed by the generalized Pauli operator ${U}_{{B}_{k},0}$. The quantum state |Φ_2_〉 is evolved as the resultant state |Φ_3_〉,which is given below:

14 $$\begin{array}{rcl}|{\Phi }_{3}\rangle  & = & {U}_{{B}_{1},0}QFT\otimes {U}_{{B}_{2},0}QFT\otimes \ldots \otimes {U}_{{B}_{k},0}QFT|{\Phi }_{2}\rangle \\  & = & {d}^{-\frac{t+1}{2}}\sum _{0\le {w}_{1},{w}_{2},\ldots ,{w}_{k} < d,\,{w}_{1}+{w}_{2}+,\ldots ,+{w}_{k}=0\,\mathrm{mod}\,d}\,|{w}_{1}+{B}_{1}\rangle |{w}_{2}+{B}_{2}\rangle \ldots |{w}_{k}+{B}_{k}\rangle \end{array}$$

**Step 7:** Each player *P*_*k*_, $k=2,3,\ldots ,t$, measures his particle $|{w}_{k}+{B}_{k}\rangle$ in computational basis and broadcasts his measurement result *w*_*k*_ + *B*_*k*_.

**Step 8:** Finally, they compute the multiplication by adding their measurement results. The multiplication of the secrets is computed as: $(a\times b)={\sum }_{k=1}^{t}\,{w}_{k}+{B}_{k}\,{\rm{mod}}\,d$.

## Correctness

In this section, we prove the correctness of our proposed protocols. We discuss the correctness proof of the secure multiparty quantum summation protocol only. The correctness proof of the secure multiparty quantum multiplication protocol is very much similar to that of the secure multiparty quantum summation protocol.

### Correctness Proof

#### lemma 1

If all players in a qualified subset ${\mathbb{S}}=\{{P}_{1},{P}_{2},\ldots ,{P}_{t}\}$ act honestly, then they can perform summation $(a+b)={\sum }_{k=1}^{t}\,{A}_{k}\,{\rm{mod}}\,d$.

#### proof 1

On Applying *QFT* and Pauli operators by each player *P*_*k*_, $k=1,2,\ldots ,t$, on his particle gives the quantum state as follows.

$$\begin{array}{rcl}|{\phi }_{3}\rangle  & = & {U}_{{A}_{1},0}QFT\otimes {U}_{{A}_{2},0}QFT\otimes \ldots \otimes {U}_{{A}_{t},0}QFT\left(\frac{1}{\sqrt{d}}\mathop{\sum }\limits_{l=0}^{d-1}\,{|l\rangle }_{1}{|l\rangle }_{2}\ldots {|l\rangle }_{t}\right)\\  & = & \frac{1}{\sqrt{d}}\mathop{\sum }\limits_{l=0}^{d-1}\,{U}_{{A}_{1},0}QFT{|l\rangle }_{1}\otimes {U}_{{A}_{2},0}QFT{|l\rangle }_{2}\otimes \ldots \otimes {U}_{{A}_{t},0}QFT{|l\rangle }_{t}\\  & = & \begin{array}{c}\frac{1}{\sqrt{d}}\mathop{\sum }\limits_{l=0}^{d-1}\,\left({U}_{{A}_{1},0}\frac{1}{\sqrt{d}}\mathop{\sum }\limits_{{m}_{1}=0}^{d-1}\,{\omega }^{{m}_{1}l}|{m}_{1}\rangle \right)\otimes \left({U}_{{A}_{2},0}\frac{1}{\sqrt{d}}\mathop{\sum }\limits_{{m}_{1}=0}^{d-1}\,{\omega }^{{m}_{2}l}|{m}_{2}\rangle \right)\\ \otimes \ldots \otimes \left({U}_{{A}_{t},0}\frac{1}{\sqrt{d}}\mathop{\sum }\limits_{{m}_{1}=0}^{d-1}\,{\omega }^{{m}_{t}l}|{m}_{t}\rangle \right)\end{array}\\  & = & \frac{1}{\sqrt{d}}\mathop{\sum }\limits_{l=0}^{d-1}\,{d}^{\frac{-t}{2}}\sum _{0\le {m}_{1},{m}_{2},\ldots ,{m}_{t} < d}\,{\omega }^{({m}_{1}+\ldots +{m}_{t})l}|{m}_{1}+{A}_{1}\rangle \otimes |{m}_{2}+{A}_{2}\rangle \otimes \ldots \otimes |{m}_{t}+{A}_{t}\rangle \\  & = & {d}^{-\frac{t+1}{2}}\sum _{0\le {m}_{1},{m}_{2},\ldots ,{m}_{t} < d}\,\mathop{\sum }\limits_{l=0}^{d-1}\,{\omega }^{({m}_{1}+\ldots +{m}_{t})l}|{m}_{1}+{A}_{1}\rangle \otimes |{m}_{2}+{A}_{2}\rangle \otimes \ldots \otimes |{m}_{t}+{A}_{t}\rangle \\  & = & {d}^{-\frac{t+1}{2}}{s}_{0}d\sum _{0\le {m}_{1},{m}_{2},\ldots ,{m}_{t} < d,\,{m}_{1}+{m}_{2}+,\ldots ,+{m}_{t}=0\,\mathrm{mod}\,d}\,|{m}_{1}+{A}_{1}\rangle \otimes |{m}_{2}+{A}_{2}\rangle \otimes \ldots \otimes |{m}_{t}+{A}_{t}\rangle \end{array}$$

Each player *P*_*k*_, $k=1,2,\ldots ,t$, measures his particle in computational basis. The compute the sum after receiving others players’ measurement results.

15 $$\mathop{\sum }\limits_{k=1}^{t}\,{m}_{k}+{A}_{k}\mathop{\equiv }\limits^{d}\mathop{\sum }\limits_{k=1}^{t}\,{m}_{k}+\mathop{\sum }\limits_{k=1}^{t}\,{A}_{k}\mathop{\equiv }\limits^{d}\mathop{\sum }\limits_{k=1}^{t}\,{A}_{k}\,{\rm{mod}}\,d$$

From Eq. (15), we get sum of the secrets $(a+b)={\sum }_{k=1}^{t}\,{A}_{k}\,\mathrm{mod}\,d$.

## Example

Here, we illustrate our proposed secure multiparty quantum summation and multiplication protocols using an example. Suppose *X* and *Y* have two secrets 4 and 2, respectively, and want to compute their sum and multiplication without revealing their secrets. The *X* and *Y* share these secrets among 7 players ${\mathscr{P}}=\{{P}_{1},{P}_{2},\ldots ,{P}_{7}\}$ and the qualified subset ${\mathbb{S}}=\{{P}_{1},{P}_{2},{P}_{3}\}$ computes the summation and multiplication. The *X* and *Y* select prime as 11. Thus, prime *d* = 11, threshold *t* = 3, and total number of players *n* = 7.

### Summation example

#### **Example**.

Consider that *X* and *Y* choose their respective polynomials $f(y)=4+2y+3{y}^{2}\,\mathrm{mod}\,11$ and $g(y)=2+3y+{y}^{2}\,\mathrm{mod}\,11$. They compute their classical shares *f*(*y*_*i*_) and *g*(*y*_*i*_) and distribute these shares among 7 players ${\mathscr{P}}=\{{P}_{1},{P}_{2},\ldots ,{P}_{7}\}$. Each player *P*_*i*_, $i=1,2,\ldots ,7$, computes $h({y}_{i})=f({y}_{i})+g({y}_{i})\mathrm{mod}\,11$. The computation of share and *h*(*y*_*i*_) are shown in Table 1. Each player *P*_*k*_, *k* = 1, 2, 3, (of qualified subset ${\mathbb{S}}=\{{P}_{1},{P}_{2},{P}_{3}\}$) computes the shadow of his share *A*_*k*_ as follows:

$${A}_{k}=h({y}_{k})\prod _{1\le j\le t,j\ne k}\,\frac{{y}_{j}}{{y}_{j}-{y}_{k}}\,{\rm{mod}}\,d$$

$${A}_{1}=4\times \left(\frac{2}{2-1}\times \frac{3}{3-1}\right)\,{\rm{mod}}\,11=1$$

$${A}_{2}=10\times \left(\frac{1}{1-2}\times \frac{3}{3-2}\right)\,{\rm{mod}}\,11=3$$

$${A}_{3}=2\times \left(\frac{1}{1-3}\times \frac{2}{2-3}\right)\,{\rm{mod}}\,11=2$$

**Table 1:** Share Computation for Summation and Multiplication.

Players		*P*_1_	*P*_2_	*P*_3_	*P*_4_	*P*_5_	*P*_6_	*P*_7_
Shares	*f*(*y*_*i*_)	9	9	4	5	1	3	0
	*g*(*y*_*i*_)	6	1	9	8	9	1	6
	*h*(*y*_*i*_) = *f*(*y*_*i*_) + *g*(*y*_*i*_)	4	10	2	2	10	4	6
	*h*′(*y*_*i*_) = *f*(*y*_*i*_) × *g*(*y*_*i*_)	10	9	3	7	9	3	0
Polynomials: *z*_*i*_(*x*)		10 + *x* + 2*x*^2^	9 + 3*x* + *x*^2^	3 + 2*x* + 2*x*^2^	7 + 2*x* + 3*x*^2^	9 + 5*x* + *x*^2^	3 + 2*x* + *x*^2^	0 + *x* + 5*x*^2^
Shares	*z*_1_(*x*_*i*_)	2	9	9	2	10	0	5
	*z*_2_(*x*_*i*_)	2	8	5	4	5	8	2
	*z*_3_(*x*_*i*_)	7	4	5	10	8	10	5
	*z*_4_(*x*_*i*_)	1	1	7	8	4	6	3
	*z*_5_(*x*_*i*_)	4	1	0	1	4	9	5
	*z*_6_(*x*_*i*_)	8	8	3	4	0	2	10
	*z*_7_(*x*_*i*_)	6	0	4	7	9	10	10
	*T*_*i*_	7	9	3	0	0	3	9

Initiator *P*_1_ computes $|{\phi }_{2}\rangle =\frac{1}{\sqrt{11}}{\sum }_{l=0}^{10}\,{|l\rangle }_{1}{|l\rangle }_{2}{|l\rangle }_{3}$ and sends the particle |*l*〉_*k*_ to each player *P*_*k*_, *k* = 2, 3. Each player *P*_*k*_ (of qualified subset ${\mathbb{S}}=\{{P}_{1},{P}_{2},{P}_{3}\}$) applies *QFT* and generalized Pauli operators *U*_1,0_, *U*_3,0_, *U*_2,0_ on his particle. Hence,

$$\begin{array}{rcl}|{\phi }_{3}\rangle  & = & {U}_{1,0}QFT\otimes {U}_{3,0}QFT\otimes {U}_{2,0}QFT\left(\frac{1}{\sqrt{11}}\mathop{\sum }\limits_{l=0}^{10}\,{|l\rangle }_{1}{|l\rangle }_{2}{|l\rangle }_{3}\right)\\  & = & \frac{1}{\sqrt{11}}\mathop{\sum }\limits_{l=0}^{10}\,{U}_{1,0}QFT{|l\rangle }_{1}\otimes {U}_{3,0}QFT{|l\rangle }_{2}\otimes {U}_{2,0}QFT{|l\rangle }_{3}\\  & = & \begin{array}{c}\frac{1}{\sqrt{11}}\mathop{\sum }\limits_{l=0}^{10}\,{U}_{1,0}\left(\frac{1}{\sqrt{11}}\mathop{\sum }\limits_{{m}_{1}=0}^{10}\,{\omega }^{{m}_{1}l}|{m}_{1}\rangle \right)\otimes {U}_{3,0}\left(\frac{1}{\sqrt{11}}\mathop{\sum }\limits_{{m}_{2}=0}^{10}\,{\omega }^{{m}_{2}l}|{m}_{2}\rangle \right)\\ \otimes {U}_{2,0}\left(\frac{1}{\sqrt{11}}\mathop{\sum }\limits_{{m}_{3}=0}^{10}\,{\omega }^{{m}_{3}l}|{m}_{3}\rangle \right)\end{array}\\  & = & \frac{1}{11}\mathop{\sum }\limits_{l=0}^{10}\,\sum _{0\le {m}_{1},{m}_{2},{m}_{3}\le 10}\,{\omega }^{({m}_{1}+{m}_{2}+{m}_{3})l}\,|{m}_{1}+1\rangle |{m}_{2}+3\rangle |{m}_{3}+2\rangle \\  & = & \frac{1}{11}\sum _{0\le {m}_{1},{m}_{2},{m}_{3}\le 10}\,\mathop{\sum }\limits_{l=0}^{10}{\omega }^{({m}_{1}+{m}_{2}+{m}_{3})l}\,|{m}_{1}+1\rangle |{m}_{2}+3\rangle |{m}_{3}+2\rangle \\  & = & 11{s}_{1}\sum _{0\le {m}_{1},{m}_{2},{m}_{3}\le 10,\,{m}_{1}+{m}_{2}+{m}_{3}=0\,\mathrm{mod}\,11}\,|{m}_{1}+1\rangle |{m}_{2}+3\rangle |{m}_{3}+2\rangle \end{array}$$

Finally, the players measure (computational basis) their particles and broadcast their respective measurement results *m*_1_ + 1, *m*_2_ + 3, *m*_3_ + 2. They get the sum by adding their measurement results as follows:

$${m}_{1}+1+{m}_{2}+3+{m}_{3}+2\mathop{\equiv }\limits^{11}{m}_{1}+{m}_{2}+{m}_{3}+6\mathop{\equiv }\limits^{11}6\,\mathrm{mod}\,11=6$$

### Example of multiplication

#### **Example**.

Similar to summation, *X* and *Y* select their respective polynomials $f(y)=4+2y+3{y}^{2}\,{\rm{mod}}\,11$ and $g(y)=2+3y+{y}^{2}\,{\rm{mod}}\,11$ and compute their respective classical shares *f*(*y*_*i*_) and *g*(*y*_*i*_). They distribute these shares among 7 players ${\mathscr{P}}=\{{P}_{1},{P}_{2},\ldots ,{P}_{7}\}$. Each player *P*_*i*_, $i=1,2,\ldots ,7$, computes $h{\prime} ({y}_{i})=f({y}_{i})\times g({y}_{i})\,{\rm{mod}}\,d$ and these $h{\prime} ({y}_{i})$ shares are shared among *n* players using new random polynomial ${z}_{i}(x)=h{\prime} ({y}_{i})+{\beta }_{1}x+{\beta }_{2}{x}^{2}+\ldots +{\beta }_{t-1}{x}^{t-1}\,{\rm{mod}}\,d$. The shares of total polynomial, denoted as *T*_*i*_, are computed by each player *P*_*i*_, $i=1,2,\ldots ,7$, using the Vandermonde matrix^18^. The computation of *T*_*i*_ is shown in Table 1. Each player *P*_*k*_, *k* = 1, 2, 3, (of qualified subset ${\mathbb{S}}=\{{P}_{1},{P}_{2},{P}_{3}\}$) computes the shadow of his shares *B*_*k*_ as follows.

$${B}_{k}={T}_{k}\prod _{1\le j\le t,j\ne k}\,\frac{{y}_{j}}{{y}_{j}-{y}_{k}}\,\mathrm{mod}\,d$$

$${B}_{1}=7\times \left(\frac{2}{2-1}\times \frac{3}{3-1}\right)\,\mathrm{mod}\,11=10$$

$${B}_{2}=9\times \left(\frac{1}{1-2}\times \frac{3}{3-2}\right){\rm{mod}}\,11=6$$

$${B}_{3}=3\times \left(\frac{1}{1-3}\times \frac{2}{2-3}\right){\rm{mod}}\,11=3$$

Initiator *P*_1_ computes $|{\Phi }_{2}\rangle =\frac{1}{\sqrt{11}}{\sum }_{r=0}^{10}\,{|r\rangle }_{1}{|r\rangle }_{2}{|r\rangle }_{3}$, sends the particle |*r*〉_*k*_ to player *P*_*k*_, *k* = 2, 3. Each player *P*_*k*_ (of qualified subset ${\mathbb{S}}=\{{P}_{1},{P}_{2},{P}_{3}\}$) applies *QFT* operation and generalized Pauli operators *U*_10,0_, *U*_6,0_, *U*_3,0_ on his particles as follows.

$$\begin{array}{rcl}|{\Phi }_{3}\rangle  & = & {U}_{\mathrm{10,0}}QFT\otimes {U}_{\mathrm{6,0}}QFT\otimes {U}_{\mathrm{3,0}}QFT\left(\frac{1}{\sqrt{11}}\mathop{\sum }\limits_{r\mathrm{=0}}^{10}\,{|r\rangle }_{1}{|r\rangle }_{2}{|r\rangle }_{3}\right)\\  & = & \frac{1}{\sqrt{11}}\mathop{\sum }\limits_{r\mathrm{=0}}^{10}\,{U}_{\mathrm{10,0}}QFT{|r\rangle }_{1}\otimes {U}_{\mathrm{6,0}}QFT{|r\rangle }_{2}\otimes {U}_{\mathrm{3,0}}QFT{|r\rangle }_{3}\\  & = & \begin{array}{c}\frac{1}{\sqrt{11}}\mathop{\sum }\limits_{r\mathrm{=0}}^{10}\,{U}_{\mathrm{10,0}}\left(\frac{1}{\sqrt{11}}\mathop{\sum }\limits_{{w}_{1}\mathrm{=0}}^{10}\,{\omega }^{{w}_{1}r}|{w}_{1}\rangle \right)\otimes {U}_{\mathrm{6,0}}\left(\frac{1}{\sqrt{11}}\mathop{\sum }\limits_{{w}_{2}\mathrm{=0}}^{10}\,{\omega }^{{w}_{2}r}|{w}_{2}\rangle \right)\\ \otimes {U}_{\mathrm{3,0}}\left(\frac{1}{\sqrt{11}}\mathop{\sum }\limits_{{w}_{3}\mathrm{=0}}^{10}\,{\omega }^{{w}_{3}r}|{w}_{3}\rangle \right)\end{array}\\  & = & \frac{1}{11}\mathop{\sum }\limits_{r\mathrm{=0}}^{10}\,\sum _{0\le {w}_{1},{w}_{2},{w}_{3}\le 10}\,{\omega }^{({w}_{1}+{w}_{2}+{w}_{3})r}\,|{w}_{1}+10\rangle |{w}_{2}+6\rangle |{w}_{3}+3\rangle \\  & = & \frac{1}{11}\sum _{0\le {w}_{1},{w}_{2},{w}_{3}\le 10}\,\mathop{\sum }\limits_{r\mathrm{=0}}^{10}{\omega }^{({w}_{1}+{w}_{2}+{w}_{3})r}\,|{w}_{1}+10\rangle |{w}_{2}+6\rangle |{w}_{3}+3\rangle \\  & = & 11{s}_{1}\sum _{0\le {w}_{1},{w}_{2},{w}_{3}\le \mathrm{10,\ }{w}_{1}+{w}_{2}+{w}_{3}\mathrm{=0}\,mod\,11}\,|{w}_{1}+10\rangle |{w}_{2}+6\rangle |{w}_{3}+3\rangle \end{array}$$

Finally, players measure (computational basis) their particle and broadcasts their measurement results *w*_1_ + 10, *w*_2_ + 6, *w*_3_ + 3. They get the multiplication by adding measurement results as follows:

$${w}_{1}+10+{w}_{2}+6+{w}_{3}+3\mathop{\equiv }\limits^{11}{w}_{1}+{w}_{2}+{w}_{3}+19\mathop{\equiv }\limits^{11}19\,\mathrm{mod}\,11=8$$

## Security Analysis

In this section, we analyze the security of our proposed secure multiparty quantum summation and multiplication protocols. We mainly focus on the security analysis of summation protocol because the security analysis of multiplication protocol is very much similar to that of summation protocol.

### Intercept-resend attack

Suppose an eavesdropper *E* intercepts the particle |*l*〉_*k*_ and measures it in the computational basis $\{|1\rangle ,|2\rangle ,\ldots ,|d-1\rangle \}$ to extract the information about the shadow of the share. The eavesdropper *E* prepares a fake particle ${\bar{l}}_{k}$ and resends it to player *P*_*k*_, $k=2,3,\ldots ,t$. If *E* performs this attack, then he can compute *l* correctly with probability $\frac{1}{d}$. But *E* cannot get any valuable information about the share of the shadow from *l*, because |*l*〉_*k*_ does not contain any valuable information about the shadow of the share.

### Collusion attack

In our proposed protocols, each player *P*_*k*_ measures his particle $|{m}_{k}+{A}_{k}\rangle$ and broadcasts his measurement result ${m}_{k}+{A}_{k}$, $k=1,2,\ldots ,t$. Thus, other players cannot obtain the shadow of the share (*A*_*k*_). Suppose the dishonest players *P*_*r*−1_ and *P*_*r*+1_ collude together to obtain other players’ shadow of the share *A*_*k*_. They cannot get any information about the shadow of the share because player *P*_1_ only sends the particles |*l*〉_*k*_ to other players; nothing else, and the particles |*l*〉_*k*_ don’t carry any valuable information about the shadow of the share. So, this attack is not possible in our proposed protocols.

### Entangle-measure attack

In this attack, the eavesdropper *E* intercepts all the particles |*l*〉_*k*_, when the initiator *P*_1_ sends the particles |*l*〉_*k*_ to player *P*_*k*_, $k=2,3,\ldots ,t$. Further, *E* selects a intercepted particle |*l*〉_*m*_ and prepares an ancillary particle |*c*〉. The *d*-level SUM operation is performed by *E* on |*l*〉_*m*_ to entangle the ancillary particle |*c*〉. The quantum state |*ϕ*_2_〉 is evolved as |*ϕ*_2_〉′, which is given below.

$$\begin{array}{rcl}|{\phi }_{2}\rangle {\prime}  & = & (SUM({|l\rangle }_{m},|c\rangle ))|{\phi }_{2}\rangle \\  & = & \frac{1}{\sqrt{d}}\mathop{\sum }\limits_{l=0}^{d-1}\,{|l\rangle }_{1},{|l\rangle }_{2},\ldots ,{|l\rangle }_{t}|l\oplus c\rangle \end{array}$$

*E* prepares another secret particle |*l*〉_*o*_ and performs *d*-level SUM operation on |*c*〉. The quantum state |*ϕ*_2_〉′ is evolved as |*ϕ*_2_〉″, which is given below.

$$\begin{array}{rcl}|{\phi }_{2}\rangle {\prime\prime}  & = & (SUM({|l\rangle }_{o},|l\oplus c\rangle ))|{\phi }_{2}\rangle \\  & = & \frac{1}{\sqrt{d}}\mathop{\sum }\limits_{l=0}^{d-1}\,{|l\rangle }_{1},{|l\rangle }_{2},\ldots ,{|l\rangle }_{t}|l\oplus l\oplus c\rangle \\  & = & |{\phi }_{2}\rangle |c\rangle \end{array}$$

The ancillary particle |*c*〉 is measured by *E* and gets the initial value |*c*〉. So, *E* concludes that the particles |*l*〉_*m*_ and |*r*〉_*r*_ are the same. Based on this conclusion, *E* assumes that all the particles |*l*〉_*k*_’s are same. Thus, *E* cannot get any information about the shadow of the share from this attack.

### Collective attack

In this attack, *E* interacts with each qudit by preparing an independent ancillary particle and jointly performs the measurement operation on all the ancillary qudit to get the shadow of the share. *E* prepares an independent ancillary particle |*c*〉 to interact with each qudit of player ${P}_{k},k=1,2,\ldots ,t$. After interacting, *E* gets the particle |*l*〉_*k*_ and jointly performs the measurement operation in the computational basis $\{|1\rangle ,|2\rangle ,\ldots ,|d-1\rangle \}$ to learn the shadow of the share. From this joint measurement, *E* cannot get any information about the shadow of the share because |*l*〉_*k*_ does not contain any information of the shadow of the share.

### Coherent attack

In coherent attack, *E* jointly interacts with all qudit of player ${P}_{k},k=1,2,\ldots ,t$, by preparing an independent ancillary particle |*c*〉 and he gets each player’s particle |*l*〉_*k*_. *E* jointly performs the measurement operation in computational basis $\{|1\rangle ,|2\rangle ,\ldots ,|d-1\rangle \}$ on each player’s particle |*l*〉_*k*_. From this measurement of particle |*l*〉_*k*_, *E* only gets *l* with probability $\frac{1}{d}$. But, *l* does not contain any valuable information about the shadow of the share. Here, *E* only knows the interacting particle |*l*〉_*k*_, nothing else. So, from this attack, *E* cannot learn the shadow of the share.

## Performance Analysis

Here, we analyze the performance of our proposed secure multiparty quantum summation and multiplication protocols and compare it with that of ten existing secure multiparty quantum summation and multiplication protocols: Du *et al.’s* protocol^4^, Chen *et al.’s* protocol^5^, Zhang *et al.’s* protocol^6^, Zhang *et al.’s* protocol^7^, Shi *et al.’s* protocol^8^, Shi’s protocol^9^, Zhang *et al.’s* protocol^10^, Liu *et al.’s* protocol^11^, Yang’s protocol^12^ and Jiao *et al.’s* protocol^13^ based on three parameters: cost, universality, and attack. The Du *et al.’s* protocol^4^ is based on multiparty computation but it has (*n*, *n*) threshold approach and its modulo is *n* + 1 and computation type is bit-by-bit. The Chen *et al.’s*^5^, Zhang *et al.s*^6^, Zhang *et al.’s*^7^ protocols are based on multiparty computation but they have (*n*, *n*) threshold approach; their modulo are 2 and computation type is bit-by-bit. The protocols of Chen *et al*.^5^ and Zhang *et al*.^6^ perform 1 Unitary operation. Both the protocols of Shi *et al*.^8^ are based on multiparty computation and computation type is secret-by-secret, but they have (*n*, *n*) threshold approach. In the Shi *et al.’s* protocols^8^, the *QFT* is applied on the first particle by initiator *Bob*_1_,who sends second particle to next player. Then, the unitary operation is performed by each player *Bob*_*i*_, *i* = 2, 3, …, *n*. Initiator *Bob*_1_ applies *QFT*^−1^ on his particle. So, the total computation and communication costs of these protocols are 1*QFT* + 1*QFT*^−1^ + (*n* − 1) unitary operations + 2 measure operation and *n*, respectively. The Shi’s protocol^9^ is based on multiparty computation but it has (*n*, *n*) threshold approach and its computation type is bit-by-bit. The Zhang *et al.’s* protocol^10^ is based on multiparty computation but it has (*n*, *n*) threshold approach with bit-by-bit computation type and modulo of 2. The total computation cost of this protocol is *n* measure operations. The Liu *et al.’s* protocol^11^ is based on multiparty computation but it has (*n*, *n*) threshold approach with bit-by-bit computation type and modulo of 2. The total computation and computation costs of this protocol is *n* and *n*, respectively. The Yang’s protocol^12^ is based on multiparty computation but it has (*n*, *n*) threshold approach. The total computation and communication costs of this protocol are *nQFT* + *n* measure operation and (*n* − 1), respectively. The Jiao *et al.’s* protocol^13^ is based on multiparty computation but it has (*n*, *n*) threshold approach with bit-by-bit computation type. The total computation and communication costs of this protocol is *n* unitary operation + *n* measure operations and *n*, respectively. However, our proposed summation and multiplication protocols are based on multiparty computation with modulo *d*, have (*n*, *n*) threshold approach, and their computation type is secret-by-secret. The total computation and communication costs of these protocols are 1*QFT* + (*t* − 1) unitary operation + *t* measure operation and (*t* − 1), respectively. So, our protocols are more secure, flexible, and practical as compared to the existing summation and multiplication protocols. The comparison of cost, universality, and attack are shown in Table 2. In this table, *IR*, *EM*, *Comt*, *Com*, s-by-s, b-by-b, Summ. and Multi. denote intercept-resend attack, entangle-measure attack, computation cost, communication cost, secret-by-secret, bit-by-bit, summation and multiplication, respectively.

**Table 2:** Comparison in terms of Cost, Universality, and Attack.

Protocols				Du^4^	Chen^5^	Zhang^6^	Zhang^7^	Shi^8^		Shi^9^	Zhang^10^	Liu^11^	Yang^12^	Jiao^13^		Proposed	
Operation				Summ.	Summ.	Summ.	Summ.	Summ.	Multi.	Summ.	Summ.	Summ.	Summ.	Summ.	Multi.	Summ.	Multi.
Performance Parameters	Costs	Comt.	*QFT*	—	—	—	—	1	1	—	—	—	*n*	—	—	1	1
			*QFT*^−1^	—	—	—	—	1	1	—	—	—	—	—	—	—	—
			Unitary Operation	—	1	1	—	*n* − 1	*n* − 1	—	—	—	—	*n*	*n*	*t* − 1	*n* − 1
			Measure Operation	—	—	—	—	2	2	—	*n*	*n*	*n*	*n*	*n*	*t*	*t*
		Com.	Message Particle	—	—	—	—	—	—	—	—	—	—	—	—	*t* − 1	*t* − 1
			Decoy Particle	—	—	—	—	*n*	*n*	—	—	*n*	*n* − 1	*n*	*n*	—	—
	Univ.	Model		(*n*, *n*)	(*n*, *n*)	(*n*, *n*)	(*n*, *n*)	(*n*, *n*)	(*n*, *n*)	(*n*, *n*)	(*n*, *n*)	(*n*, *n*)	(*n*, *n*)	(*n*, *n*)	(*n*, *n*)	(*t*, *n*)	(*t*, *n*)
		Modulo		*n* + 1	2	2	2	*N*	*N*	—	2	2	*d*	*N*	*N*	*d*	*d*
		Qubits		—	—	—	—	$\lceil {log}_{2}^{d}\rceil n$	$\lceil {log}_{2}^{d}\rceil n$	$2\lceil {log}_{2}^{d}\rceil n$	—	2	—	—	—	—	—
		Type of Computation		b-by-b	b-by-b	b-by-b	b-by-b	s-by-s	s-by-s	b-by-b	b-by-b	b-by-b	s-by-s	b-by-b	b-by-b	s-by-s	s-by-s
	Attacks	IR		—	*Yes*	*Yes*	*Yes*	*Yes*	*Yes*	—	*Yes*	*Yes*	*Yes*	*Yes*	*Yes*	*Yes*	*Yes*
		Collusion		—	*Yes*	*No*	*No*	—	—	—	*Yes*	—	*Yes*	—	—	*Yes*	*Yes*
		EM		—	—	*Yes*	—	*Yes*	*Yes*	—	—	*Yes*	*Yes*	*Yes*	*Yes*	*Yes*	*Yes*
		Collective		—	—	—	—	—	—	—	—	—	—	—	—	*Yes*	*Yes*
		Coherent		—	—	—	—	—	—	—	—	—	—	—	—	*Yes*	*Yes*

## Conclusion

In this paper, we have presented the hybrid (*t*, *n*) threshold quantum protocols for multiparty summation and multiplication. They have better communication and computation costs as the type of computation is secret-by-secret. They can resist the quantum attacks, i.e., intercept-resend, entangle-measure, collusion, collective, and coherent. No player cannot get other players’ private information. Our protocols are more secure, flexible, and practical as compared to the existing summation and multiplication protocols. Further, they possess all the benefits (i.e., use qudit instead of qubit, do not pass any secret information through the transmitted particles, do not perform entanglement measurement, and quantum attacks are not possible) of the existing secure multiparty quantum summation and multiplication.
